# Adipose gene expression related to body condition score and individual variations in hospitalized cats

**DOI:** 10.1038/s41598-025-22397-1

**Published:** 2025-11-05

**Authors:** Tomoyuki Sugiyama, Fumie Shimokawa, Kazutoshi Sugiyama, Takashi Kobayashi, Yusuke Yamashita, Masayuki Funaba, Masaru Murakami

**Affiliations:** 1 Laboratory of Molecular Biology, Azabu University School of Veterinary Medicine, Sagamihara, 252-5201 Japan; 2 Sugiyama-Animal-Hospital, Shizuoka, 424-0068 Japan; 3 Kobayashi Animal Hospital, Nagano, 380-0816 Japan; 4 Aoi Animal Hospital, Shizuoka, 420-0076 Japan; 5 Division of Applied Biosciences, Kyoto University Graduate School of Agriculture, Kyoto, 606-8502 Japan

**Keywords:** Cats, Adipose tissue, Body condition score, Mitochondrial respiration, Gene expression, Gene expression, Animal physiology

## Abstract

Obesity is a risk factor for various diseases. The prevalence of obesity is increasing in cats. The present study aimed to characterize adipose gene expression to obtain basic information for obesity prevention in feline adipose tissues. Visceral fat, subcutaneous fat, or both fat depots were collected from 81 hospitalized cats. The multivariate analysis of covariance revealed that sex and IGF-1 were involved in regulating body condition score (BCS), showing adiposity in both fat depots. BCS was positively and negatively regulated by expression levels of inflammation-related genes in visceral fat, respectively. In addition, subcutaneous *Lep* expression positively regulated BCS. Positive correlations between gene expression levels were generally detected within the same fat depot, whereas expression levels of genes in visceral fat were less related to those in subcutaneous fat, except for the same gene. Expression levels of *Ucp1* were most variable among individuals in visceral fat but not in subcutaneous fat. The extent of individual variability on expression levels in cats was similar to that in the previous results using dogs. Genes related to mitochondrial respiration and uncoupling were relatively variable among individuals. The present study suggests that feline energy metabolism in adipose tissues is finely tuned in a fat depot-dependent manner. In addition, genes related to mitochondrial respiration and uncoupling may be a target for the control of systemic energy status because they can be potentially regulated by every individual.

**Supplementary Information:**

The online version contains supplementary material available at 10.1038/s41598-025-22397-1.

## Introduction

The prevalence of obesity, which results from hyperplasia and hypertrophy of white adipocytes, is increasing every year in cats^1^. More than 40% of domesticated cats have been estimated to be overweight and obese worldwide^2^. In addition, overweight in cats has already reached up to 63% in New Zealand in 2007^3^. Obesity is associated with the prevalence of hepatic lipidosis, ophthalmic disorders, non-allergic skin diseases, digestive system disorders, lameness, heart diseases, insulin resistance, and urinary system disorders in cats^2^. Therefore, similar to most mammals, prevention and treatment of obesity are important to maintain quality of life in cats.

In addition to white adipocytes accumulating excess energy as fat, brown and beige adipocytes are known^4–6^. Although the cell lineages are distinct among these three adipocytes^7^, the bone morphogenetic protein (BMP) pathway commonly stimulates differentiation into white/brown/beige adipocytes in humans and mice^7,8^. Unlike white adipocytes, brown and beige adipocytes consume the chemical energy through the expression of uncoupling protein (UCP) 1 that uncouples the oxidation of fatty acids and sugars with ATP production^4–6^. The energy expenditure in brown and beige adipocytes affects systemic energy balance: obesity was induced by the removal of brown adipocytes in mice^9^. In addition, the activity of brown/beige adipocytes was negatively related to the body mass index (BMI), showing adiposity in humans^10^. These results suggest that adiposity is regulated by the balance of activities among white adipocytes, brown adipocytes, and beige adipocytes. Thus, brown and beige adipocytes are useful targets for the prevention of obesity and obesity-related metabolic disturbances in humans^4,11^.

Adipose tissue not only stores energy but also produces many bioactive molecules, adipokines^12–14^. Adipokines are positively and negatively involved in maintaining systemic health through the regulation of insulin responsiveness and inflammation^12–14^. Adipokine production is dependent not only on adiposity but also on the location of the fat depots in humans and mice^12–14^. However, the expression pattern varies between animal species^15–18^, and the differences in gene expression profiles between visceral fat and subcutaneous fat are not clear in cats.

As the first step to enable the control of feline adiposity, the present study aimed to characterize adipose gene expression in cats. We evaluated the gene expression of the BMP pathway components, adipokines, and genes related to adipocyte differentiation and function, insulin signaling, brown adipogenesis and uncoupling, inflammation, and cell division in the visceral fat and subcutaneous fat of hospitalized cats.

## Results

### Genes affecting BCS in hospitalized cats

**Fig. 1:**
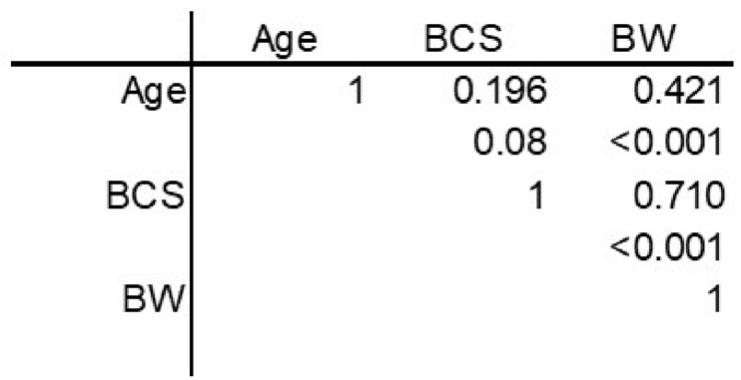
Relationship between age, BCS, and body weight of hospitalized cats. Upper: *r* value, Lower: *P* value. *n* = 81.

 We first evaluated the relationship between age, BCS, and body weight in cats (Fig. 1). These criteria were mutually interrelated, suggesting that obesity and overweight increase with age (Fig. 1). Specific disease was not linked to aberrant expression of the gene (data not shown). The multivariate analysis of covariance was performed to evaluate factors affecting BCS (Table 1). Sex was involved in BCS in both types of fat: male strongly affected BCS. Expression levels of *Igf1* were also involved in BCS in both fat depots: *Igf1* positively and negatively regulated BCS in visceral fat and subcutaneous fat, respectively. Inflammation-related *Tyrobp*, *Cd53*, *Plek*, and *Mmp9* expressions were involved in BCS in visceral fat, respectively. In addition, expression levels of *Actr2a*, *Cidea*, and *Pck1* regulated BCS in visceral fat. In contrast, subcutaneous *Lep* expression positively regulated BCS.

**Table 1:** Multivariable linear regressions on BCS.

Fat depot	Variable	Estimated parameter	SE	*P* value		Log worth	VIF
Visceral fat	Intercept	1.747	0.231	< 0.001			
	Sex	0.793	0.142	< 0.001		6.10	1.19
	*Tyrobp*	-0.496	0.112	< 0.001		4.11	2.10
	*Cidea*	-0.357	0.088	< 0.001		3.77	2.65
	*Cd53*	0.471	0.139	0.001		2.87	4.38
	*Actr2a*	0.308	0.102	0.004		2.40	1.16
	*Pck1*	0.209	0.070	0.004		2.36	1.66
	*Mmp9*	-0.052	0.018	0.006		2.20	1.72
	*Igf1*	0.199	0.089	0.03		2.25	2.25
	*Plek*	0.120	0.072	0.10		3.39	3.39
	Overall r^2^: 0.610						
Subcutanoue fat	Intercept	2.370	0.321	<0.001			
	*Igf1*	-0.272	0.093	0.005		2.30	1.12
	Sex	0.663	0.250	0.01		1.99	1.15
	*Lep*	0.168	0.066	0.01		1.87	1.07
	Overall r^2^: 0.298						

### Changes in adipose gene expression in overweight cats

We compared expression levels of adipose genes between normal cats with BCS3 and overweight cats with BCS4 or BCS5 (BCS4/5, Tables 2 and 3). Expression levels of *Adipoq* and *Tnfa* were lower and higher in cats with BCS4/5 than in cats with BCS3 in visceral fat (Table 2). Insulin signal-related *Irs1* and brown adipogenesis-related *Cidea*, *Pgc1a*, and *Prdm16* expressions were lower in visceral fat in overweight cats than in normal cats. In contrast, visceral *Plek* and *Gadd45b* expressions were higher in overweight cats than in normal cats. In subcutaneous fat, insulin signal-related *Igf1* and *Irs1* expressions were lower in overweight cats than in normal cats (Table 3). In contrast, expression levels of *Lep* and *Angptl4* were higher in overweight cats than in normal cats.

**Table 2:**
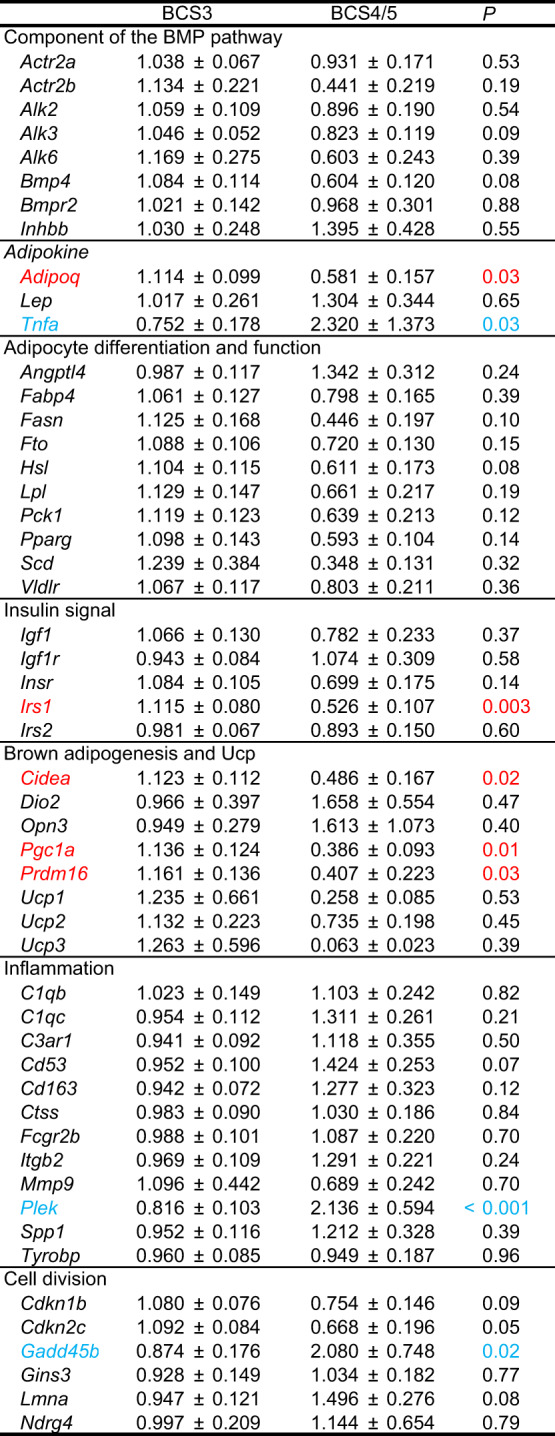
Gene expression in visceral fat. Mean ± SE. Average expression in visceral fat of all cats is set at 1. BCS3 (n=48), BCS4/5 (n=9).Genes of which expression level was significantly higher and lower in the cat with BCS3 than in the cat with BCS4/5 are shown in red and blue, respectively.

**Table 3:**
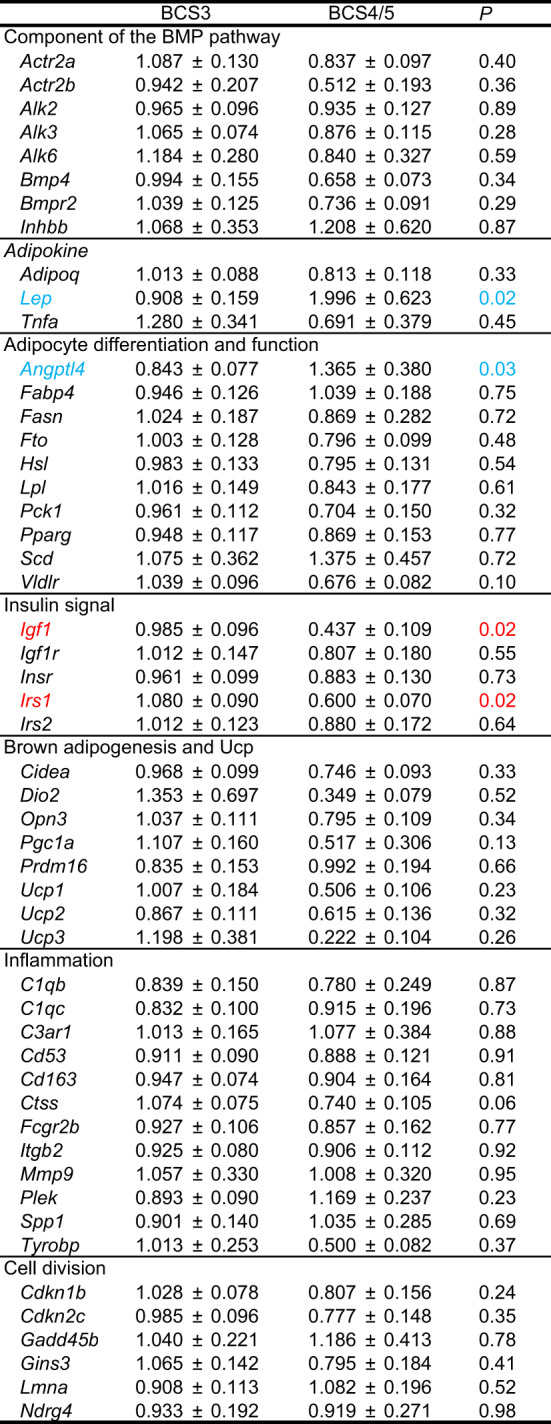
Gene expression in subcutaneous fat.Mean ± SE. Average expression in visceral fat of all cats is set at 1. BCS3 (n=44), BCS4/5 (n=9).Genes of which expression level was significantly higher and lower in the cat with BCS3 than in the cat with BCS4/5 are shown in red and blue, respectively.

### Relationship of gene expression levels between visceral fat and subcutaneous fat 

**Fig. 2:**
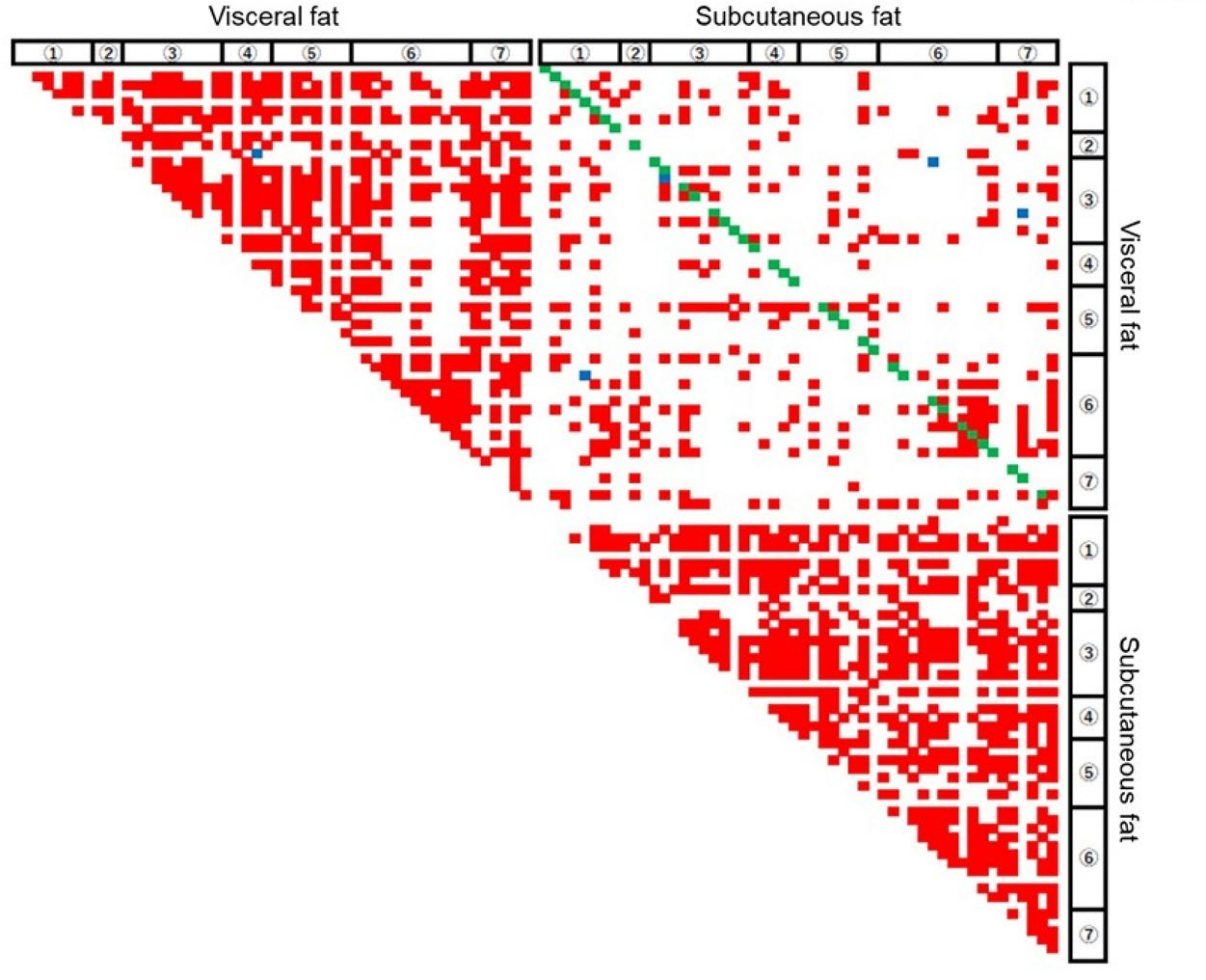
Summarized relationship between expression levels of genes in visceral fat and subcutaneous fat of hospitalized cats. A statistically significant relationship of the correlation coefficient is shown in color; red and blue: positive and negative relationship except for the same gene, respectively; green: positive relationship on the same gene. *n* = 48. Gene group: (1) component of the BMP pathway, (2) adipokine, (3) adipocyte differentiation and function, (4) insulin signal, (5) brown adipogenesis and *Ucp*, (6) Inflammation, and (7) cell division.

The relationship of gene expression levels is shown in Fig. 2. Generally, positive relationships between gene expression levels were detected within the same fat depot: the close relationships were detected in visceral fat and subcutaneous fat. In contrast, the gene levels in visceral fat were less correlated with those in subcutaneous fat, except for the same gene. Thirty-seven genes among the examined 52 genes exhibited significant and positive correlations between expression levels in visceral fat and those in subcutaneous fat.

**Fig. 3:**
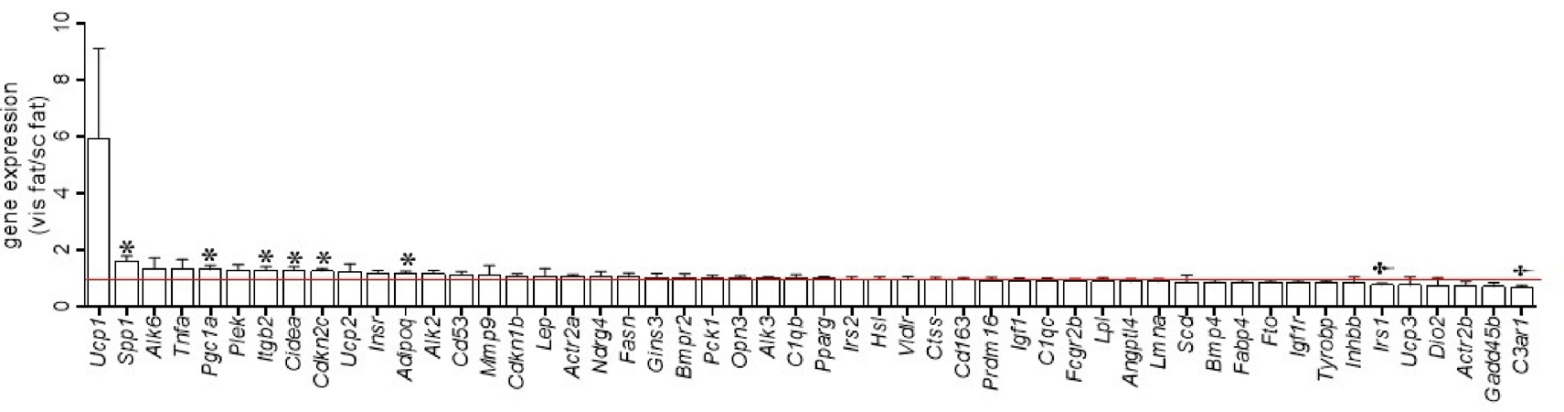
The ratio of expression levels of genes in visceral fat to those in subcutaneous fat in hospitalized cats. * and †: Significantly higher and lower expression in visceral fat as compared with expression level in subcutaneous fat at *P* < 0.05, respectively. The red line indicates 1, which means equal levels between visceral fat and subcutaneous fat.

We also evaluated differences in gene expression levels between visceral fat and subcutaneous fat (Table 4). Expression levels of 7 genes, i.e., *Actr2a*, *Alk6*, *Adipoq*, *Pgc1a*, *Ucp1*, *Spp1*, and *Cdkn2c*, were significantly higher in visceral fat than in subcutaneous fat, and 2 genes (*Irs1* and *Gadd45b*) exhibited more expression in subcutaneous fat than in visceral fat. Because both visceral fat and subcutaneous fat were obtained in 48 cats, we also compared gene expression levels in both fat depots by the ratio of expression in visceral fat to that in subcutaneous fat (Fig. 3). Similar to the above results, expression levels of *Spp1*, *Pgc1a*, *Cdkn2c*, and *Adipoq* were higher and those of *Irs1* were lower in visceral fat than in subcutaneous fat. In addition, expression levels of *Itgb2* and *Cidea* were higher in visceral fat, whereas *C3ar1* expression was higher in subcutaneous fat (Fig. 3). Generally, genes related to brown adipogenesis and *Ucp* were highly expressed in visceral fat (Table 4; Fig. 3). The high and low ratio of expression levels in visceral fat to those in subcutaneous fat (Fig. 3) was not necessarily consistent with statistical significance. These results suggest the existence of genes with large individual variations.

**Table 4:**
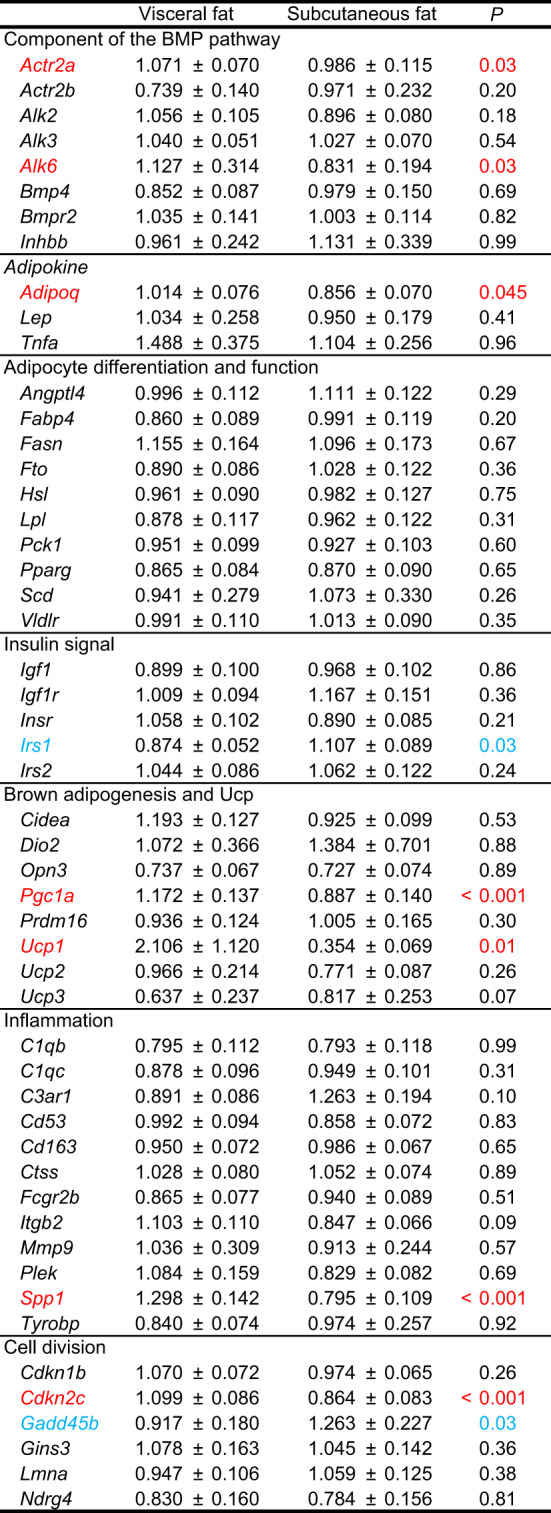
Gene expression in fat depots of cats.Mean ± SE. Abdominal fat (n=63), subcutaneous fat (n=67).Genes of which expression level was significantly higher and lower in abdominal fat than subcutaneous fat are shown in red and blue, respectively.

**Table 5:** Summary of the descriptive GO term names of the functional clusters on genes expression levels with large variation.

Fat depot	Category	Enrichment score
Visceral fat	Oxidative phosphorylation uncoupler activity	3.68
	Cytokine-receptor interaction	2.16
Subcutanoue fat	TGF-β family signaling	2.82

### Individual variations of gene expression levels in adipose tissues

**Fig. 4:**
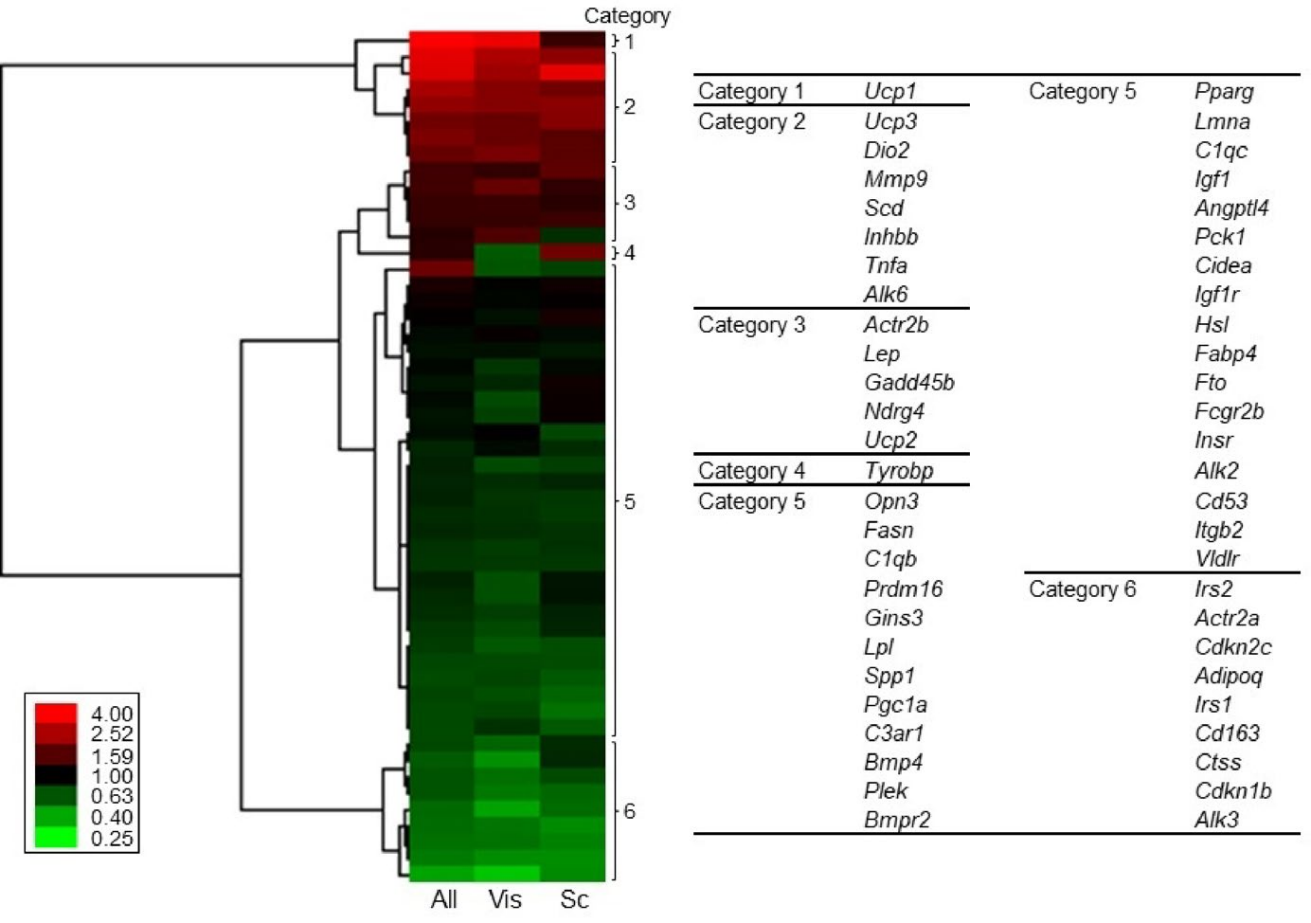
Individual variations of gene expression levels in all fat, visceral fat, and subcutaneous fat. Heatmap shows the CV value of the gene expression levels in cats, and according to a correlation coefficient, genes were categorized into 6 groups.

To categorize individual variations of gene expression levels, we created a heatmap showing coefficient of variance (CV) value of the gene expression levels in hospitalized cats (Fig. 4). According to a correlation coefficient between the CV values, genes were divided into 6 groups: large variations in visceral fat but not in subcutaneous fat (category 1), large variations both in visceral fat and subcutaneous fat (category 2), moderate variations in both types of fat (category 3), large variation in subcutaneous fat but not in visceral fat (category 4), relatively stable (category 5) and more stable (category 6) in both fats.

**Fig. 5:**
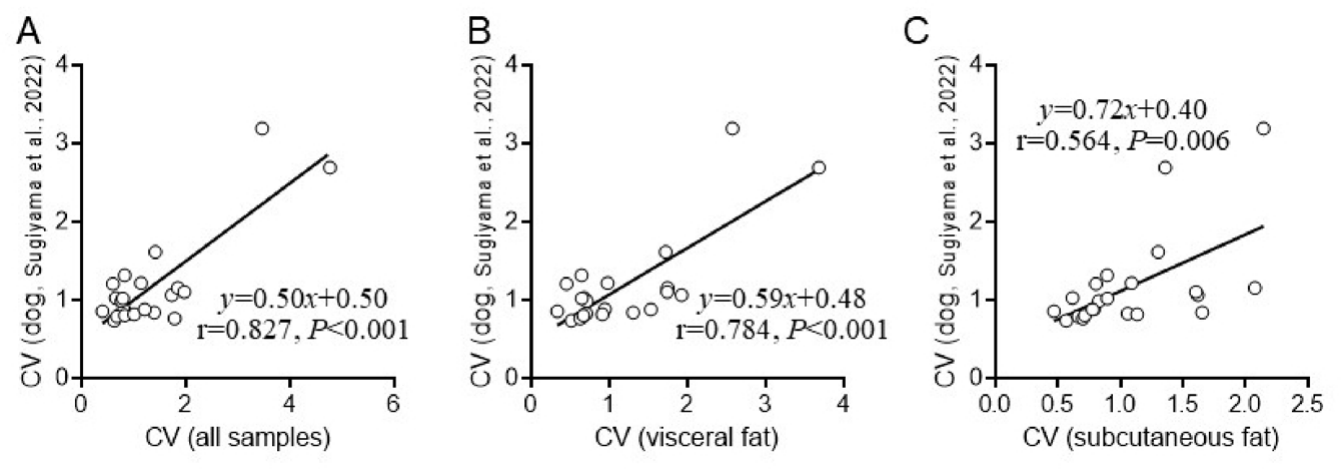
Comparisons of CV values of gene expression levels between cats in this study and dogs in our previous study (Sugiyama et al., 2022). The CV values in all samples (**A**), visceral fat samples (**B**), and subcutaneous fat samples (**C**) were compared with those in fat samples of dogs (Sugiyama et al., 2022).

The individual variation of adipose *Ucp1* expression was highest in cats (Fig. 4). Our previous study also showed a large variation between individuals on *Ucp1* expression levels in dogs^19^. Thus, we compared the CV values in cats with those in dogs from our previous study^19^ (Fig. 5). We found positive correlations of the CV values between cats and dogs.

We further inferred the biological pathway exhibiting large individual variations from the top 10 genes with high CV values by DAVID, a bioinformatic tool (Table 5). The oxidative phosphorylation and uncoupler, as well as cytokine-receptor interaction, were estimated to be more variable between individuals in visceral fat. In contrast, genes related to the TGF-β family signaling showed large individual variations in subcutaneous fat.

## Discussion

Here, we characterized factors related to obesity in hospitalized cats. The multivariate analysis of covariance revealed that sex and expression levels of adipose *Igf1* is involved in adiposity in both fat depots: BCS was higher in male cats than female cats. The expression levels of *Igf1* in visceral fat positively related to BCS, whereas BCS score was negatively related to the subcutaneous *Igf1* (Table 1). The IGF1 pathway potently promotes body growth, but hepatic IGF1 is a major source in whole body^20^. The physiological significance of involvement of adipose *Igf1* expression in adiposity is currently unknown. Among inflammation-related genes, visceral *Tyrobp*, *Cd53*, *Plek*, and *Mmp9* expressions were involved in the regulation of BCS (Table 1). PLEK and TYROBP are markers of inflammation^21,22^: Salivary PLEK levels were higher in patients with chronic periodontitis^21^. The *Plek* mRNA levels were increased by treatment with IL-1β (an inflammatory cytokine) or lipopolysaccharides (an inducer of inflammation) in fibroblasts^21^. The expression of *Tyrobp* was increased during inflammation, and activation of microglial-like cells was blocked by knockdown of the *Tyrobp* gene^22^. The multivariate analysis of covariance also revealed that BCS was positively and negatively regulated by expression levels of *Plek* and *Tyrobp*, respectively, in visceral fat but not subcutaneous fat. Previous studies showed that expression levels of *Plek* and *Tyrobp* in subcutaneous fat were higher in obese human patients than in lean humans^23^. Although a close relationship between obesity and inflammation is well-established^24^, the (path-)physiological significance of PLEK and TYROBP in obesity-related inflammation is unclear yet in cats.

The increase in the plasma level of LEP, a pro-inflammatory adipokine^25^, has been shown in obese patients^13^. Consistent with the findings, the multivariate analysis of covariance indicated that BCS was positively regulated by expression levels of *Lep* in feline subcutaneous fat. In fact, subcutaneous *Lep* expression was higher in cats with BCS4/5 than in cats with BCS3 (Table 3). LEP has an activity to depress food intake, but elevated LEP levels do not reduce appetite in obese humans, indicating LEP resistance^13^. The detailed role of LEP should be clarified in obese cats in the future.

In addition to subcutaneous *Lep* expression, *Adipoq* and *Tnfa* expressions in visceral fat were related to obesity: *Adipoq* and *Tnfa* expressions were lower and higher in obese cats, respectively (Table 2). Similar changes in the adipose expression of *Adipoq* and *Tnfa* related to obesity were shown in cats^26,27^. Insulin resistance is induced in obese humans and mice^28^. ADIPOQ and TNFA increase insulin sensitivity and insulin resistance, respectively, through modulation of phosphorylation and activation of IRS-1 (a molecule determining insulin sensitivity^29^^30,31^. In the present study, expression levels of *Irs-1* in visceral fat was also lower in overweight cats (Table 2). Insulin resistance in obese cats^32^ may be induced not only through down-regulation of *Irs-1* expression but also through inactivation of IRS-1 resulting from modulation of *Adipoq* and *Tnfa* expressions.

The present study also evaluated the differential expression of *Adipoq*, *Lep*, and *Tnfa* different between visceral fat and subcutaneous fat in cats (Table 4; Fig. 3): the expression of *Adipoq* in visceral fat was higher than that in subcutaneous fat. Expression levels of *Lep* and *Tnfa* were comparable between visceral fat and subcutaneous fat. These results contrast with the previous results in humans and mice. Lihn et al.^17^ showed lower expression levels of *Adipoq* in visceral fat in humans irrespective of adiposity. Expression levels of *Lep* were lower in visceral fat than in subcutaneous fat in lean and obese humans^15,18^. Furthermore, Zha et al.^18^ revealed lower *Tnfa* expression in visceral fat in obese humans but not in lean humans. The differential expression of adipokines depending on animal species may reflect the unique regulation of energy metabolism in adipose tissues.

The present study also indicated that expression levels of genes related to brown/beige adipocytes (*Ucp1*, *Pgc1a*, and *Cidea*, Table 4; Fig. 3) were higher in visceral fat than in subcutaneous fat, suggesting that brown/beige adipocytes are present more in feline visceral fat. These results are consistent with the supposed distribution of brown/beige adipocytes in cattle^33^ but contrast to that in mice, showing higher expression of brown adipocyte-related genes in subcutaneous fat than in visceral fat^34^. Expression levels of brown adipogenesis-related *Cidea*, *Pgc1a*, and *Prdm16* were lower in cats with BCS4/5 than in cats with BCS3 (Table 2). These results suggest that brown/beige adipocytes are present in fewer numbers in obese cats. A decrease in the number at their major location of brown/beige adipocytes is likely to lead to insufficient energy expenditure, resulting in the onset of obesity.

The present study revealed close relationships between genes within the same fat depot. These results suggest that gene expression is globally regulated in a fat depot. Cell-specific transcription is accomplished by an epigenetic program^35^. The epigenetic regulation, such as histone acetylation and methylation as well as DNA methylation, may lead to global regulation in a fat depot-dependent gene expression. Future studies should be conducted to clarify detailed mechanisms.

Feline hepatic lipidosis is the most common form of liver disease diagnosed in North America^36^ and induces secondary impairment of liver function and intrahepatic cholestasis^37,38^. An imbalance between the influx of free fatty acids derived from adipose tissues, *de novo* synthesis of fatty acids, the rate of β-oxidation of fatty acids, and the disposal of hepatic triglycerides via secretion of very low-density lipoproteins has been suggested as a pathological mechanism underlying feline hepatic lipidosis^38,39^. In a retrospective study, the mortality within 60 days after hospitalization was 38% in cats with hepatic lipidosis^40^. Obesity is a risk factor for feline hepatic lipidosis^41,42^. Therefore, efficient utilization of free fatty acids in activated brown and beige adipocytes may be effective in preventing feline hepatic lipidosis. The present study provides basic information on the block of liver damage through the improvement of adiposity in cats.

The present study indicated a gene-dependent magnitude of individual variation (Fig. 4). The extent of the variations of gene expression levels was comparable between cats in this study and dogs in our previous study^19^ (Fig. 5). These results suggest that the strength of regulation of every gene expression in fat depot is similar between cats and dogs, which suggest that genes exhibiting a small variation in their expression levels are rigorously regulated. In contrast, expression levels of genes with a large variation might be affected by various factors that are variable between individuals, including nutritional and metabolic status. The genes with large individual variations were estimated to be related to oxidative phosphorylation uncoupler activity in visceral fat in cats (Table 5). In particular, *Ucp1* was the most variable gene in cats and dogs^19^. Large individual variations of *Ucp1* were also detected in cattle^33^. UCP1 is a responsible molecule in energy expenditure in brown/beige adipocytes^43,44^. Clarification of factors affecting feline *Ucp1* expression enables the control of mitochondrial respiration activity and thermogenesis in brown/beige adipocytes, which may lead to the prevention of obesity and obesity-related diseases in cats.

The present study has some limitations. The information of dietary history was unavailable. Because the nutritional condition affects gene expression and adiposity, the dietary history should be recorded in future. The present study used BCS as an index of obesity. BCS is a practical but semi-quantitative index. In addition, there was no cat with BCS1 in this study, and sex distribution is unbalanced (71 females and 10 males) because of the study design. These limitations make us to be difficult to assess gene expression across the full spectrum of adiposity. Furthermore, because the fat samples were generally collected during spay or neutering, 73% of cats were below 1 year of age, but the age range was wide (0.33–18.00 years). Therefore, it should be noted that the data from cats with different physiological stages were analyzed.

## Methods

### Cats

All animal care and experiments were approved by the Institutional Animal Care and Use Committee of Azabu University (200109-2). All animal experiments were conducted following approved guidelines. The study is reported in accordance with ARRIVE guidelines (https://arriveguidelines.org). Eighty-one client-owned cats, 0.33–18.00 years of age (mean ± SD: 2.38 ± 4.00, 71 female and 10 male cats) were used. A detailed profile of each cat, including the reason for hospitalization for surgery, is listed in Supplementary Table S1. A total of 130 fat depot samples were collected in 3 private animal clinics: 68 cats (116 samples), 9 cats (9 samples), and 4 cats (5 samples). Cats were intramuscularly treated with medetomidine or with xylazine and ketamine to sedate, followed by anesthetization with isoflurane inhalation for spay or neutering. In the case of diseased cats, alfaxalone was used as an anesthetic induction agent. Fat depots (visceral fat, *n* = 63, and subcutaneous fat, *n* = 67) were collected during the surgery. The omental fat was collected during the laparotomy as visceral fat, and subcutaneous fat was obtained from the abdominal area. Cat no. 69 suffered from hydronephrosis resulting from urolithiasis, and the perirenal fat was also obtained during the surgery. The testis is usually removed through an incision in the scrotum for castration, except for cryptorchidism. Because cat no. 1, 67, 73, and 78 were diagnosed as cryptorchidism, the testis was removed by the laparotomy, and the omental fat was obtained. As the result, sex distribution (71 female and 10 male) was uneven. Both fat depots were obtained from 48 cats. The use of fat depots for the experiment was informed to the clients by veterinarians, and consent was obtained. Each fat depot was immersed in the RNAlater solution (ThermoFisher, Waltham, MA, USA) for RNA stabilization, according to the manufacturer’s protocol.

BCS ranging 1–5 was judged by veterinarians: (1) very thin, (2) underweight, (3) ideal, (4) overweight, and (5) obese. BCS and body weight ranged from 2 to 5 (3.0 ± 0.7) and 1.90–7.75 kg (3.23 ± 1.20), respectively. They were brought to three private animal clinics and were judged to be clinically healthy (69 cats) or diseased (12 cats). The major reason for admission was surgery for neutering (spaying: 65 cats, castration: 4 cats). Three cats (cat no. 2, 71, and 72) exhibited BCS5 (highly obese). Cat no. 71 may suffer from hepatic lipidosis because the cat exhibited anorexia and vomiting. In addition, plasma levels of alanine aminotransferase, alkaline phosphatase, and total bilirubin were higher than the reference value. The other two cats (cat no. 2 and 72) did not exhibit anorexia and were diagnosed with urolithiasis.

### RNA isolation and RT-quantitative (q) PCR

RNA isolation and real-time quantitative PCR (RT-qPCR) were performed as described previously^19^. The oligonucleotide sequences of the primers for RT-qPCR are presented in Supplementary Table S2. The expression level of genes was normalized against that of hypoxanthine phosphoribosyltransferase 1 (*Hprt1*), and the average in all samples was set at 1.

### Heatmap

To create a heat map of individual variations of gene expression, we set the CV value of the expression levels of the gene. The CV values in all fat, visceral fat, and subcutaneous fat were subjected to hierarchical cluster analysis using Euclidean distances and complete linkage grouping with Cluster 3.0. Hierarchical cluster analysis was visualized in green and red using the Java TreeView software (http://jtreeview.sourceforge.net/). The threshold for grouping was set at a correlation coefficient of 0.9 in the dendrogram, and the aim was to group the data points into as few groups as possible.

### Functional category analysis

Functional categories enriched in the top 10 variable genes between individuals were identified by use of the database for annotation, visualization, and integrated discovery (DAVID^45^, as shown previously^46^.

### Statistical analyses

Multicollinearity analysis to evaluate the gene expression affecting BCS was performed using JMP Pro 18 (SAS Institute, Cary, NC, USA), and forward-backward stepwise variable selection was chosen. The dependent variables included BCS, and the independent variables included sex (female: 1, male: 2), age, and the relative expression levels of genes. All variables remaining in the model were significant at *P* < 0.15. Furthermore, the reciprocal relationship between the relative expression levels of genes was investigated using Pearson’s correlation coefficient. As for the ratio of expression levels in visceral fat to those in subcutaneous fat, the 95% CI value was calculated. *P* < 0.05 was considered significant.

## Supplementary Information

Below is the link to the electronic supplementary material.

Supplementary Material 1

Supplementary Material 2

Supplementary Material 3

## Data Availability

All data included in this study are available upon reasonable request by contact with the corresponding author.
